# Glucose Concentration in Regulating Induced Pluripotent Stem Cells Differentiation Toward Insulin-Producing Cells

**DOI:** 10.3389/ti.2024.11900

**Published:** 2024-01-18

**Authors:** Chencheng Wang, Shadab Abadpour, Petter Angell Olsen, Daxin Wang, Justyna Stokowiec, Simona Chera, Luiza Ghila, Helge Ræder, Stefan Krauss, Aleksandra Aizenshtadt, Hanne Scholz

**Affiliations:** ^1^ Department of Transplant Medicine, Institute for Surgical Research, Oslo University Hospital, Oslo, Norway; ^2^ Hybrid Technology Hub, Center of Excellence, University of Oslo, Oslo, Norway; ^3^ Department of Immunology and Transfusion Medicine, Oslo University Hospital, Oslo, Norway; ^4^ Department of Molecular Medicine, Institute of Basic Medical Sciences, University of Oslo, Oslo, Norway; ^5^ Department of Clinical Science, University of Bergen, Bergen, Norway; ^6^ Department of Pediatrics, Haukeland University Hospital, Bergen, Norway

**Keywords:** stem cell-derived beta cells, mitochondria, glucose, stem cell differentiation, induced pluripotent stem cells

## Abstract

The generation of insulin-producing cells from human-induced pluripotent stem cells holds great potential for diabetes modeling and treatment. However, existing protocols typically involve incubating cells with un-physiologically high concentrations of glucose, which often fail to generate fully functional IPCs. Here, we investigated the influence of high (20 mM) versus low (5.5 mM) glucose concentrations on IPCs differentiation in three hiPSC lines. In two hiPSC lines that were unable to differentiate to IPCs sufficiently, we found that high glucose during differentiation leads to a shortage of NKX6.1+ cells that have co-expression with PDX1 due to insufficient *NKX6.1* gene activation, thus further reducing differentiation efficiency. Furthermore, high glucose during differentiation weakened mitochondrial respiration ability. In the third iPSC line, which is IPC differentiation amenable, glucose concentrations did not affect the *PDX1*/*NKX6.1* expression and differentiation efficiency. In addition, glucose-stimulated insulin secretion was only seen in the differentiation under a high glucose condition. These IPCs have higher KATP channel activity and were linked to sufficient *ABCC8* gene expression under a high glucose condition. These data suggest high glucose concentration during IPC differentiation is necessary to generate functional IPCs. However, in cell lines that were IPC differentiation unamenable, high glucose could worsen the situation.

## Graphical Abstract

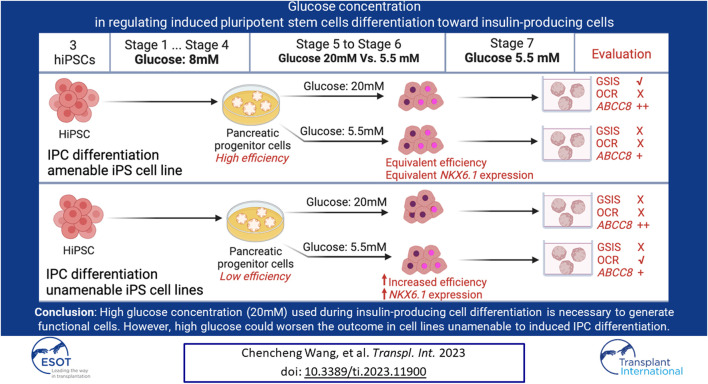

## Introduction

Cellular therapy as a treatment option for type 1 diabetes (T1D) may benefit from improving current protocols for generating insulin-producing cells (IPCs) from human-induced pluripotent stem cells (hiPSC). Existing studies have shown the possibility of using hiPSC for differentiating functional IPCs *in vitro* [1]. Glucose is an important energy source and a primary physiological regulator of insulin biosynthesis and secretion for IPCs [2]. IPCs differentiation from early reports [3, 4] to state-of-the-art protocols has relied on non-physiological high glucose concentrations during the differentiation. These differentiation protocols applied a glucose concentration of 8–15 mM until the pancreatic progenitor (PP) stage (stage 4), followed by incubation in a differentiation medium containing 20–25.5 mM glucose in stage 5/6 but often reduced in the final maturation stage. The above protocols endowed IPCs with functional properties, but showed metabolic abnormalities, lower oxidative phosphorylation levels, and an immature mitochondria morphology [5–9]. It is known that high glucose causes adverse effects on human primary islets [10, 11], but why high glucose is needed during IPC differentiation has not been well studied.

To gain insights into the impact of different glucose concentrations in regulating IPCs differentiation from hiPSCs, we studied three hiPSC lines from different sources in our model by following a seven-stage protocol with minor modifications [3, 12]. In which, after reaching the pancreas progenitor stage (stage 4), a low (5.5 mM), non-physiological high (20 mM) and an insufficient energy condition mimicked by 5–6, 2-deoxy-D-glucose (2-DG) were applied to continue the differentiation until maturation stage. We analyzed the IPC differentiation efficiency, gene expression profiles, and co-localization of transcription factors such as NKX6.1 and PDX1. Furthermore, glucose’s impacts on IPCs functionalities, including glucose-stimulated insulin secretion (GSIS), calcium flux, and oxygen consumption.

## Materials and Methods

### Human iPSC Differentiation and Human Islets

The information on hiPSC and human islets was displayed in Supplementary Tables S1, S2. Human primary islets were maintained in CMRL 1066 (Corning, 15-110-CV) supplemented with 5% human AB serum (PAN-Niotech GmbH), L-Glutamine, 1% penicillin/streptomycin, 10 mM HEPES (all from Gibco) on ultra-low attachment plates (Corning, CLS3261). The hiPSCs were cultured in E8 Medium (Gibco, A1517001), and confirmed to be mycoplasma-free. The differentiation was done using the seven stages protocol [3] with modification [12]. On day 1 of the suspension culture, Rho Kinase inhibitor Y27632 (StemCell Technologies, 72304) was added to prevent cell death (Supplementary Table S3).

### Flow Cytometry and Immunofluorescence Analysis

Primary and secondary antibodies were incubated for 45–60 min at RT or overnight at 4°C (Supplementary Table S4). LSR-II or LSRFortessa (BD Biosciences) and FlowJo (v.10.8.1, Treestar) were used for flow cytometry analysis. Images were taken with Leica TCS SP8 microscope and analyzed with Fiji (v.2.3.0). Trainable WEKA segmentation plugin [13] was used to identify particles in the images, and Fiji ROI manager was used to map the co-localization.

### Glucose-Stimulated Insulin Secretion (GSIS)

Cell clusters were hand-picked into cell culture inserts (Merck, CLS3414) placed in 24-well cell culture plates. Cells were equilibrated in Krebs-Ringer buffer (KRB) with 1.67 mM glucose for 1 h at 37°C before being subjected to sequential 1-h incubation of 1.67 mM (Low), 20 mM (High) and 1.67 mM (Low) glucose, and then 20 mM glucose with 30 mM KCl in KRB for 30 min. Dynamic GSIS was performed using a perfusion system (Suprafusion 1000, BRANDEL). Sixty hand-picked cell clusters and 20 hand-picked human islets were used for each channel. Samples were collected every 6 min, and insulin was measured using human insulin ELISA kits (Mercodia, 10-1113-10).

### Oxygen Consumption and Calcium Flux Analysis

The seahorse XFe24 analyzer (Agilent) was used to measure oxygen consumption, as described [14]. 40–60 cell clusters were picked for analysis. The oxygen consumption values were normalized to the baseline. Calcium imaging was performed as previously described [4]. Stage 7+ cell clusters were attached to 1:100 diluted Geltrex-coated chambers (Ibidi, 80827), incubated at 37°C overnight, and labeled with 20 µM Fluo4-AM (Molecular Probes, F14201). Time series images were acquired every 15 s with Leica TCS SP8 and analyzed with Fiji. The Fiji plugin Register Virtual Stack Slices [15] was used for image alignment.

### Insulin Contents, Lactate, and Glucose Measurement

Cells were lysis by CellTiter-Glo 3D Cell Viability Assay Kit (Promega, G9681) and measured with human insulin ELISA kits. The insulin content was normalized to the total protein. The lactate and glucose levels in the cell culture supernatant were measured by a blood gas analyzer (Radiometer, ABL800 FLEX). Glucose uptakes were calculated by glucose supplemented in medium minus glucose left in the daily cell culture supernatant. Uptake ratios were calculated from glucose uptakes divided by glucose supplemented.

### Western Blot and qRT-PCR

Total proteins and RNA were isolated with TRIzol (Invitrogen, 15596026). Protein samples were separated with 8% Midi Protein Gels (Invitrogen, WG1001A). Primary and HRP-conjugated secondary antibodies were incubated at 4°C overnight or RT for 1 h. Images were developed in the ChemiDoc MP System (Bio-Rad). Semi-quantification analysis was conducted by using Fiji. A cDNA Reverse Transcription Kit (Applied Biosystems, 4368814) was used for cDNA synthesis. PowerUp SYBR Green (Applied Biosystems, A25780) based RT-PCR was performed with Viia 7 RT-PCR system (Applied Biosystems). Gene expression was normalized to Tbp (TATA box binding protein) and human islets’ gene expression profile. Heatmap was analyzed and plotted with Heatmapper [16] with the average linkage clustering method, and Manhattan clustering algorithms were selected to compute distances.

### Mitochondrial Contents and Membrane Potential Analysis

Stage 6 cells were dissociated as single cells and incubated with 100 mM MitoTracker DeepRed (Invitrogen, M22426). To analyze insulin + subpopulation, samples were incubated with the anti-insulin antibody and analyzed with LSRFortessa. Undifferentiated hiPSC without dye or only with secondary antibodies was performed as the negative control. For mitochondrial membrane potential analysis, 2 µM JC-1 dye was incubated with cells for 30 min. Cells incubated with 4 µM CCCP were set up as negative control. For mtDNA/gDNA ratio analysis, the total DNA was extracted using the Mammalian Genomic DNA Miniprep Kits (Sigma, G1N70) and determined with SYBR Green-based qPCR. Primers are listed in Supplementary Table S5.

### Statistical Analysis

Data were plotted as mean ± SD unless otherwise indicated. A two-tailored Student’s t-test was used for the analysis of statistical significance by using GraphPad Prism 8.4.0 software. Sample size (*n*) is specified in each figure caption and indicates biological replicates unless otherwise noted.

## Results

### Differentiation Under a High Glucose Concentration Medium Decreased NKX6.1/PDX1 Co-Localization in Non-Pancreatic Preferable Cell Lines

**FIGURE 1:**
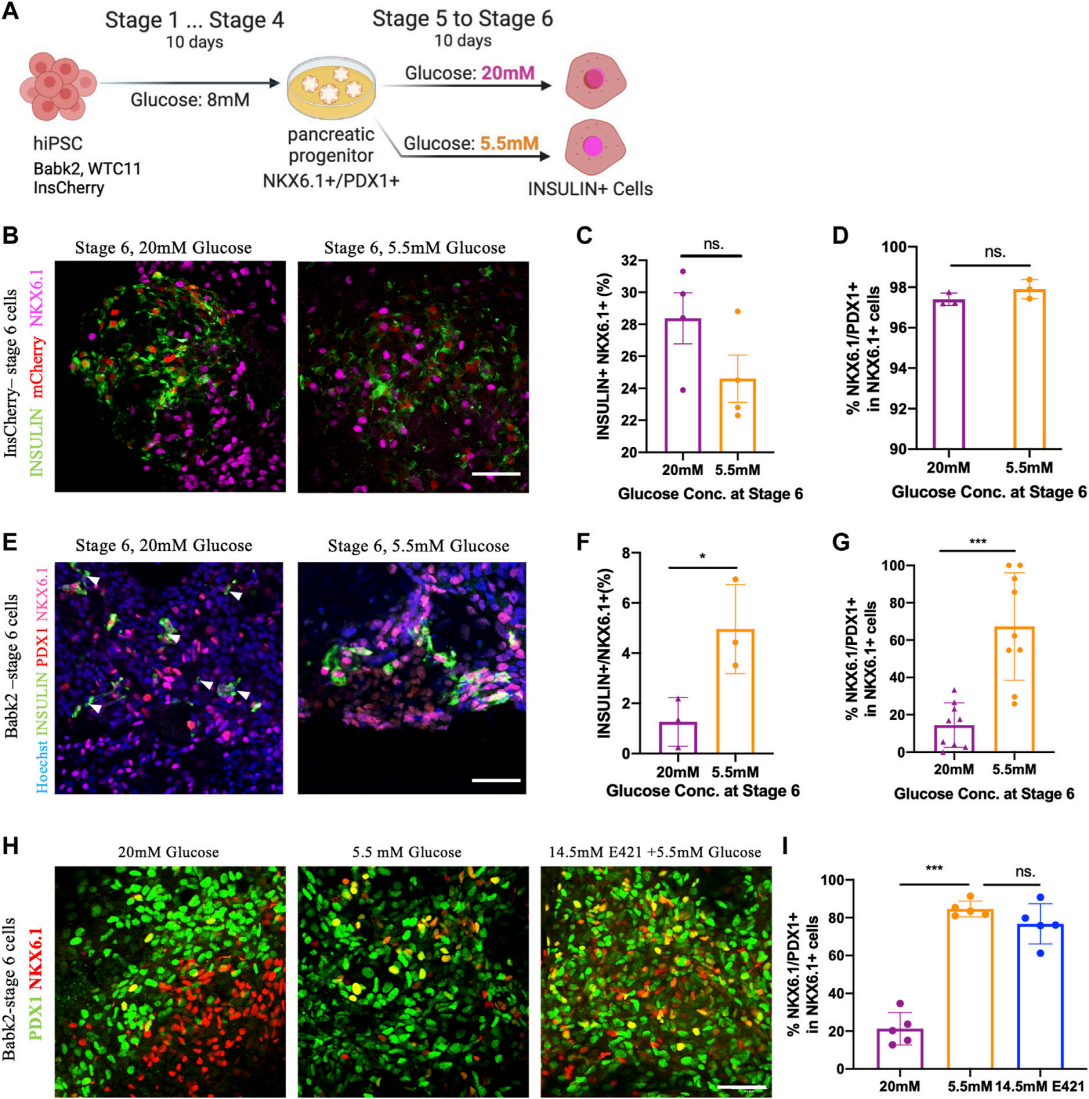
High glucose concentration differentiation decreased NKX6.1/PDX1 co-localization in non-pancreatic preferable cell lines. **(A)** Outline of the experiment design: cells after the PP stage (stage 4) were cultured in stage 5 and 6 medium containing 5.5 mM or 20 mM of glucose for 10 days. Created with BioRender.com. **(B)** Immunostaining of INSULIN and NKX6.1 in InsCherry-stage 6 cells, mCherry also representing insulin + cells. **(C)** Flow cytometry quantification of InsCherry-stage 6 cells staining for INSULIN and NKX6.1, *n* = 4. **(D)** PDX1/NKX6.1 co-localization percentage among NKX6.1+ cells in InsCherry-stage 6 cells, *n* = 3. **(E)** Immunostaining for Babk2-stage 6 cells, the white arrowheads indicated the insulin + cells which have no NKX6.1 detected. **(F)** Flow cytometry analysis of Babk2-steg6 cells staining for INSULIN and NKX6.1, *n* = 3. **(G)** Immunostaining quantification analysis of PDX1 and NKX6.1 co-localization percentage among NKX6.1+ cells in Babk2-stage 6 cells, *n* = 9. **(H)** Immunostaining for Babk2-stage 6 cells for PDX1 and NKX6.1. **(I)** Immunostaining quantification analysis of NKX6.1/PDX1 co-localization percentage among NKX6.1+ cells in Babk2-stage 6 cells. E421 (mannitol), 14.5 mM mannitol supplemented in 5.5 mM glucose medium. *n* = 5. Scale bars represent 50 μm; ns. Non-significant; *p < 0.05, ***p < 0.001 by unpaired two-way t-tests.

To study the impact of glucose concentration during IPC differentiation, three hiPSC lines derived PP cells (stage 4) continued to differentiate in high glucose (20 mM) and low glucose (5.5 mM) medium till stage 6 (Figure 1A). The cell line differentiation efficiency till stage 4 was exanimated (Supplementary Figure S1A). The InsCherry iPSC line differentiated stage 6 cells (InsCherry-stage 6 cells) under high glucose showed a stronger insulin signal, but the differentiation efficiency was unaffected (Figures 1B, C; Supplementary Figure S2A). PDX1 and NKX6.1 are critical transcription factors to maintain beta cell identity [17]. It has been shown PDX1-/NKX6.1+ cells can also continue to differentiate into IPCs [18]. However, low levels of *Pdx1* accompany IPCs’ dysfunction in experimental models of glucotoxicity and diabetes [19]. Therefore, we quantified the NKX6.1/PDX1 subcellular co-localization in InsCherry-stage 6 cells. Over 95% of NKX6.1+ cells were co-localized with PDX1 among all NKX6.1+ cells (Figure 1D).

In contrast to InsCherry-stage 6 cells, the Babk2 and WTC cell lines demonstrated a lower IPC differentiation efficiency (Supplementary Figure S1A) and had less than 10% IPCs at stage 6 (Supplementary Figures S2B, C). The lower efficiency obtained from Babk2 and WTC11 cell lines is consistent with previous reports showing variations among cell lines [3, 4]. Interestingly, a low glucose differentiation of the Babk2 and WTC11 cell lines at stages 5–6 resulted in a higher percentage of IPCs at stage 6 (Figure 1F; Supplementary Figures S2B, C). In Babk2-stage 6 cells, the quantitative analysis of NKX6.1 subcellular co-localization showed less than 30% of NKX6.1+ cells co-localized with PDX1 when cells were differentiated in a high glucose medium (Figure 1G). A similar effect of high glucose impact in WTC11-stage 6 cells was observed (Supplementary Figures S2D, E). Furthermore, we frequently observed babk2-stage 6 INSULIN + cells with undetectable NKX6.1 through immunostaining under high glucose differentiation (Figure 1E; Supplementary Figure S2F). Significantly more glucagon+/insulin + cells can be observed in Babk2-stage 6 cells differentiated under high glucose (Supplementary Figures S3A, B). Reduced proliferation is an important hallmark of mature beta cells [9]. The cell cycle distribution in the cells differentiated under different glucose conditions has no significant difference, but the InsCherry-stage 6 cells had a significantly higher proportion of phase G1 cells compared to Babk2-stage 6 cells differentiated under high glucose (Supplementary Figures S3C, D).

To determine whether the effect of abnormal co-expression of PDX1/NKX6.1 depends on glucose concentration but not osmotic pressure, we supplemented 14.5 mM mannitol in 5.5 mM glucose medium at stages 5–6 to mimic an equivalence osmotic pressure. In Babk2-stage 6 cells, there was no significant difference in NKX6.1/PDX1 co-localization between the low glucose and osmotic control group (Figures 1H, I). However, Babk2-stage 6 cells differentiated under high glucose constantly had significantly less NKX6.1/PDX1 co-localization among NKX6.1+ cells (Figure 1I). The results indicated the loss of PDX1 expression among NKX6.1+ cells was because of high glucose applied during differentiation in non-pancreatic preferable cell lines.

The recently published protocols decreased the glucose concentration from above 20 mM at the maturation stages [6, 8], in which the maturation stage was comparable with stage 7 and stage 7+ in this study. Therefore, we investigated whether decreasing the glucose concentration at the maturation stages could rescue the reduced NKX6.1/PDX1 co-localization (Supplementary Figure S3E). The stage 6 cells differentiated under high glucose did not show significant differences in NKX6.1/PDX1 co-localization after 7 days of incubation, regardless of the glucose concentrations applied (Supplementary Figures S3F, G). Furthermore, the cells showed less than 20% of NKX6.1/PDX1 co-localization on average, suggesting that the loss of co-localization may be irreversible *in vitro* by lowering the glucose concentration for non-pancreatic preferable cell line differentiation.

The analysis of Babk2 and WTC11 cell lines suggested a negative impact of differentiating IPCs in a non-physiological high glucose medium, leading to a lower IPC differentiation efficiency and less co-localization of NKX6.1/PDX1, in which high glucose shows a long-term negative impact on differentiation efficiency *in vitro*. Of note, the above effect may have been overlooked by focusing on improving IPC differentiation efficiency by amenable PSC lines, such as the InsCherry cell line.

### The High Glucose Slows Down *NKX6.1* Gene Activation in Non-Pancreatic Preferable Cell Lines

**FIGURE 2:**
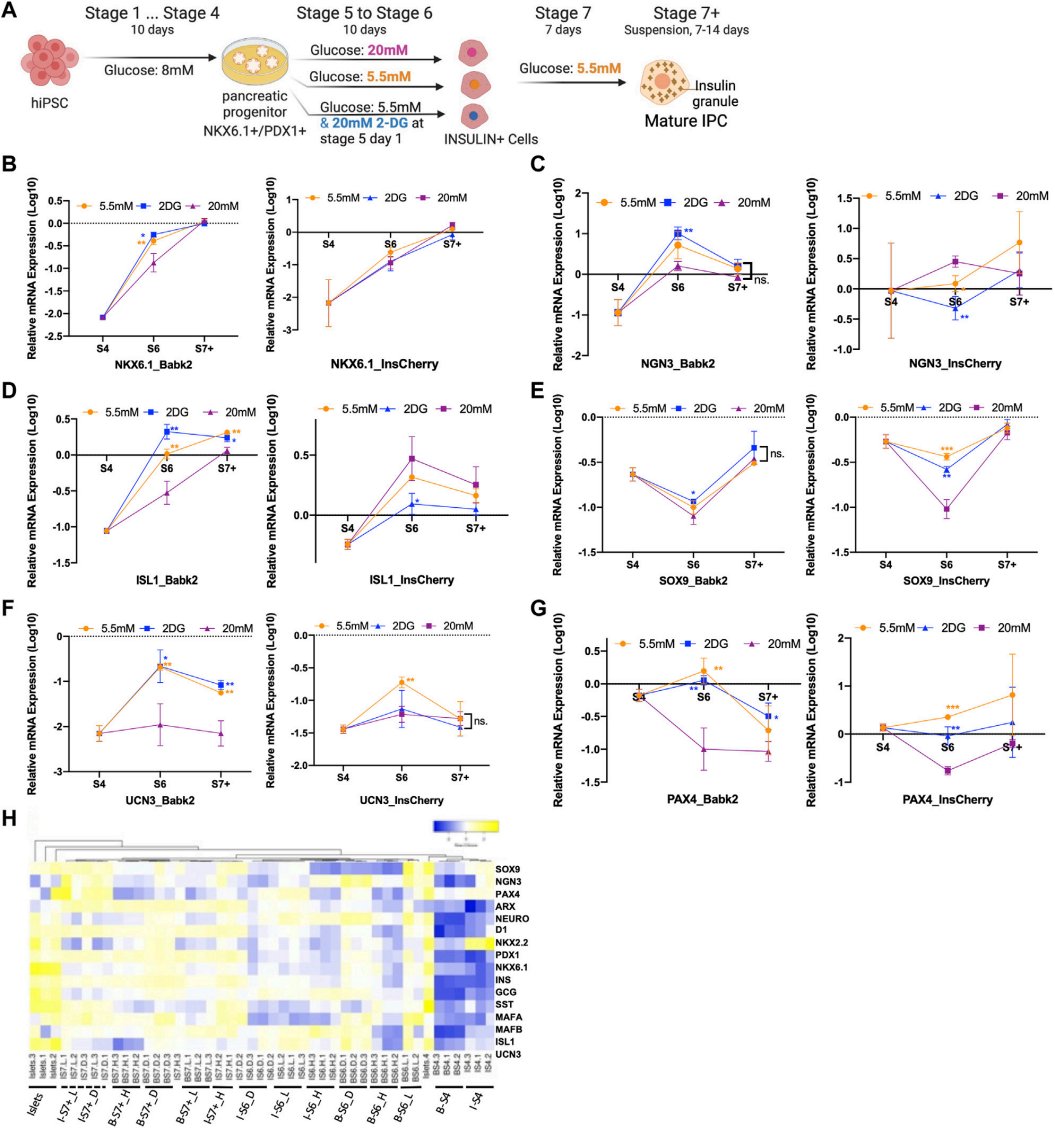
Gene expression profile analysis. **(A)** Outline of the experiment design: cells after the PP stage (stage 4) were cultured in stage 5 and 6 medium containing 5.5 mM glucose with/without 20 mM 2DG, and 20 mM of glucose for 10 days, the cells were then entered to stage 7 and stage 7+ medium containing 5.5 mM glucose respectively. **(B–G)** Real-time PCR gene expression analysis of Babk2 and InsCherry cell line differentiated cells at different stages (*n* = 3). Data were normalized to *TBP* and then human islets (*n* = 4), “*Y*-axis = 0” representing the mean value of each gene expression in human islets. **(H)** Heatmap of 15 endocrine-related genes expression at different stages. “I”, InsCherry cell line; “B”, Babk2 cell line; “L”, differentiation medium containing 5.5 mM low glucose; “H”, differentiation medium containing 20 mM high glucose; “D”, 20 mM 2-DG was added on the first day in the differentiation medium containing 5.5 mM glucose. 2-DG, 2-deoxy-D-glucose; S4, stage 4; S6, stage 6; S7+, cell clusters at stage 7+; ns, non-significant; *p < 0.05, **p < 0.01, ***p < 0.001 by unpaired two-way t-tests.

To determine how different energy statuses could impact the IPCs differentiation during stages 5 and 6, 2-DG, a competitive inhibitor of glucose phosphorylation, was adopted to mimic a fasting condition during differentiation (Figure 2A). Given that prolonged incubation with 2-DG induced severe cell death (data not shown), 20 mM of 2-DG was added in the first 24 h in the stage 5 medium containing 5.5 mM glucose. The three iPSC lines derived stages 4, 6, and 7+ cells were collected for a pancreatic lineage specification gene expression analysis.

The gene expression analysis showed a significantly lower *NKX6.1* expression in Babk2-and WTC11-stage 6 cells under a high glucose condition (Figure 2B, left), and no significant difference was detected in *PDX1* expression at stage 6 (Supplementary Figures S4A, B). In addition, no significant difference in *NKX6.1* expression was found in InsCherry-stage 6 cells (Figure 2B, right), consistent with the immunostaining image quantification that shows no significant difference in NKX6.1/PDX1 co-localization (Figure 1D). The results indicate that NKX6.1/PDX1 co-localization reduction is due to insufficient *NKX6.1* activation under the high glucose differentiation at stage 6. However, the *NKX6.1* expression shows no significant difference for Babk2-stage 7+ cells, which revealed that the high glucose might slow down *NKX6.1* activation.

### Pancreatic Lineage Gene Expression Analysis Revealed a Delayed *NGN3* Activation in Non-Pancreatic Preferable Cell Lines

*NGN3* is critical in the specification of endocrine cell development [20]. Babk2-stage 4 cells had a lower *NGN3* expression than InsCherry-stage 4 cells (Figure 2C). At stage 6, upregulation of *NGN3* expression was only detected in the Babk2 cell line. The *NGN3* upregulation was more significant in a low glucose condition and was highly elevated in a nutrient-deficient condition mimicked by supplementing 2-DG in a 5.5 mM glucose medium. The applied protocol in this study conducted stage 5 cells as pancreatic endocrine precursors and stage 6 cells as immature beta cells, whereas *NGN3* should be activated in the early days of stage 5 [3]. Therefore, we concluded that *NGN3* has a delayed activation pattern in non-pancreatic preferable cell lines, such as the Babk2 in this study.

Although a possibly delayed *NGN3* activation was found in non-pancreatic preferable cell lines, a significantly higher *NGN3* expression was found in Babk2 (Figure 2C) and WTC11 (Supplementary Figure S4B) cell lines at stage 6 under low glucose. The Babk2 and WTC11 cell lines in low glucose differentiation had a higher IPC differentiation efficiency (Figure 1F; Supplementary Figure S4B), indicating that a low glucose condition could increase *NGN3* activation and thus improve IPCs differentiation efficiency in non-pancreatic preferable cell lines. This result provided insight into further optimizing the IPCs differentiation protocol, especially for cell lines such as Babk2 or WTC11.

### Varied Glucose Concentrations Are the Primary Cause of the Different Gene Expression Profiles

*Islet-1 (ISL-1)* is critical for ensuring the differentiation of pancreatic endocrine progenitors [21]. Its expression in Babk2 (Figure 2D, left) and WTC11 (Supplementary Figure S4B) cell lines benefited from low glucose and maintained a higher expression level until the end. In contrast, the glucose concentration had the opposite impact on *ISL1* expression in the InsCherry cell line (Figure 2D, right). Several other gene expressions displayed similar levels between cell lines. For instance, *SRY-Box Transcription Factor 9 (SOX9)*, specific toward the non-endocrine cell lineage differentiation at the later stage of pancreatic development [22], had a lower expression at stage 6 under high glucose differentiation, suggesting that the high glucose concentration during differentiation inhibits non-endocrine cell development (Figure 2E). However, this short-term impact was not maintained till the end. Other beta cell maturation markers, including *Urocortin 3 (UCN3)* and *Paired box 4 (PAX4)*, had higher expression levels in a low glucose condition in both Babk2 and InsCherry differentiated cells (Figures 2F, G).

The gene expression correlation analysis revealed that Babk2 and InsCherry differentiated cells at the different stages were clustered separately (Figure 2H). The stage 7 cells formed as a separate group and were closer to human primary islets, meaning that the cells were successfully differentiated towards islet-like populations. Notably, the cells differentiated under low and high glucose conditions were clustered separately at each stage. Furthermore, Babk2-stage 6 cells in the osmotic control group clustered closer with the cells in the low glucose group but separated from cells differentiated under high glucose (Supplementary Figure S4C). Thus, variations in glucose concentrations during differentiation appeared to be a primary cause for the different gene expression profiles.

### High Glucose Differentiation Improves Functional IPC Development

**FIGURE 3:**
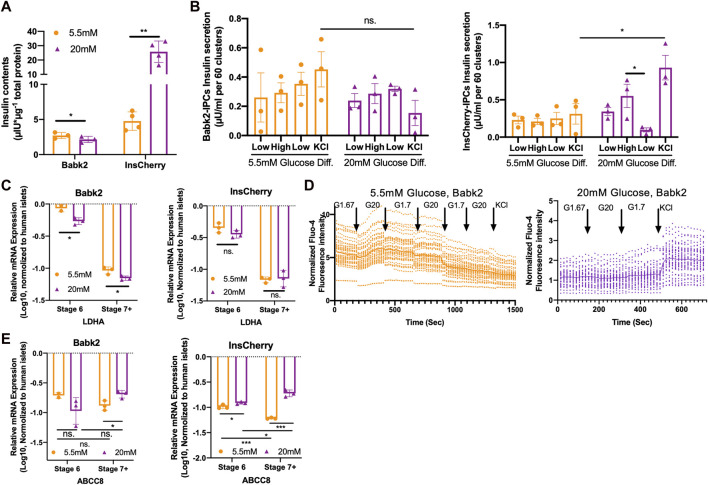
High glucose concentration helps functional IPCs development. **(A)** Total insulin contents in Babk2-stage 7+ cells (*n* = 3) and InsCherry-stage 7+ cells (*n* = 4) differentiated under low (5.5 mM) and high (20 mM) glucose conditions. Insulin contents values were normalized to total protein. **(B)** GSIS of Babk2-stage 7+ cells (left) and InsCherry-stage 7+ cells (right) differentiated under low (5.5 mM) and high (20 mM) glucose conditions, *n* = 3. **(C)** *LDHA* expression analysis for Babk2 (left) and InsCherry (right) cell line differentiated cells at different stages (*n* = 3), data were normalized to *TBP* and human primary islets (*n* = 4). “*Y*-axis = 0” represents the mean value of *LDHA* expression in human islets. **(D)** The calcium flux analysis for Babk2-stage 7+ cells differentiated under low (5.5 mM, left) and high (20 mM, right) glucose conditions. Over 30 independent cells were traced and analyzed in each group, and the data were normalized to background light intensity. The solid line shows average Fluo-4 intensity. **(E)** *ABCC8* expression analysis for Babk2 (left) and InsCherry (right) cell line differentiated cells at different stages (*n* = 3), data were normalized to *TBP* and then human islets (*n* = 4), “*Y*-axis = 0” representing the mean value of *ABCC8* expression in human islets. Ns. Non-significant, *p < 0.05, **p < 0.01, ***p < 0.001 by unpaired two-way t-tests.

To investigate how the different glucose concentrations at stages 5 and 6 affect the functionality of the differentiated cells, we decreased the glucose concentration from 20 to 5.5 mM at stage 7 in the following studies (Figure 2A). The Babk2-stage 7+ cells had lower total insulin contents under high glucose differentiation (Figure 3A), which could be a consequence of its lower IPC differentiation efficiency in high glucose. In contrast, InsCherry-stage 7+ cells had significantly higher total insulin contents in the cells differentiated under high glucose (Figure 3A).

The Babk2-stage 7+ cells did not show an activated insulin secretion in response to glucose stimulation, which is consistent with the InsCherry cell line differentiated under a low glucose condition (Figure 3B). Of note, the Babk2-Stage 7+ cells derived from high glucose differentiation showed a decreased but not significant KCl-mediated insulin secretion (3B, left), which might be because of the significantly lower IPCs yielding (Figure 1F) and insulin contents (Figure 3A). In contrast, the InsCherry differentiated under 20 mM glucose achieved an increased insulin secretion upon high glucose stimulation, and then the insulin secretion significantly decreased in response to the following incubation in a low glucose environment (Figure 3B, right). InsCherry-stage 7+ cells differentiated under high and low glucose conditions can respond to 20 mM glucose stimulation in dynamic GSIS evaluation but were not comparable with human primary islets (Supplementary Figures S5A, B).

### High Glucose Concentration During Differentiation Efficiently Suppressed *LDHA* Gene Expression

We observed that the cell culture medium changed to bright-yellow under a high glucose condition at stages 5–6. Therefore, we hypothesized that the cells under a high-glucose differentiation had higher glycolytic activity and thus produced more lactate acid, which decreased the pH of the cell culture medium. The lactate measurements in daily medium supernatant supported that the cells differentiated under high glucose had a dramatically higher lactate production than in low-glucose conditions. In addition, the cells differentiated under low glucose have higher glucose utilization percentages (Supplementary Figures S5C, D).

Lactate dehydrogenase A (LDHA) is a so-called “disallowed” gene in beta cells due to its deficient expression in healthy beta cells [23]. We found that the *LDHA* was efficiently suppressed alongside the differentiation (Figure 3C). However, significantly less *LDHA* mRNA was detected in Babk2-derived cells under a high glucose condition (Figure 3C), suggesting a high glucose concentration could induce more efficient *LDHA* expression suppression in non-pancreatic preferable cell lines.

### High Glucose Differentiation Improves KATP Channel Formation Through the Upregulation of *ABCC8* Gene Expression

Increased glucose levels lead to beta cell membrane depolarization, causing calcium ions influx and eventually triggering insulin secretion [4]. Therefore, we monitored the intracellular calcium flux in Babk2-stage 7+ cell clusters at the single-cell level. The Babk2-stage 7+ cells differentiated at low glucose responded to sequential glucose challenge by increasing intracellular calcium but failed to have increased intracellular calcium in response to cell membrane depolarization induced by 30 mM KCl (Figure 3D, left). In contrast, Babk2-stage 7+ cells differentiated under high glucose revealed few changes in Fluo-4 fluorescence intensity towards glucose stimulation but had an increased calcium influx after cell membrane depolarizing (Figure 3D, right).

To investigate why stage 7+ cells have a different calcium ions influx profile, we analyzed metabolism-related gene expressions under different glucose conditions (Supplementary Figure S6). Notably, the *ABCC8*, which encodes Sulfonylurea receptor-1 (SUR1) protein as a part of the KATP channel in regulating insulin secretion [24], was found to be significantly upregulated under high glucose differentiation (Figure 3E). In contrast, *ABCC8* expression decreased during stage 6 to stage 7+ under low glucose. Taken together with the lower expression of *ABCC8* and its failed activation during the maturation stages under low glucose, our results revealed the important role of glucose in regulating *ABCC8* expression, and a low glucose differentiation failed to trigger the KATP channel’s efficient forming due to the inadequate activation of *ABCC8* expression.

### High Glucose Differentiation Mediates the Inhibition of the Hippo Signaling Pathway

**FIGURE 4:**
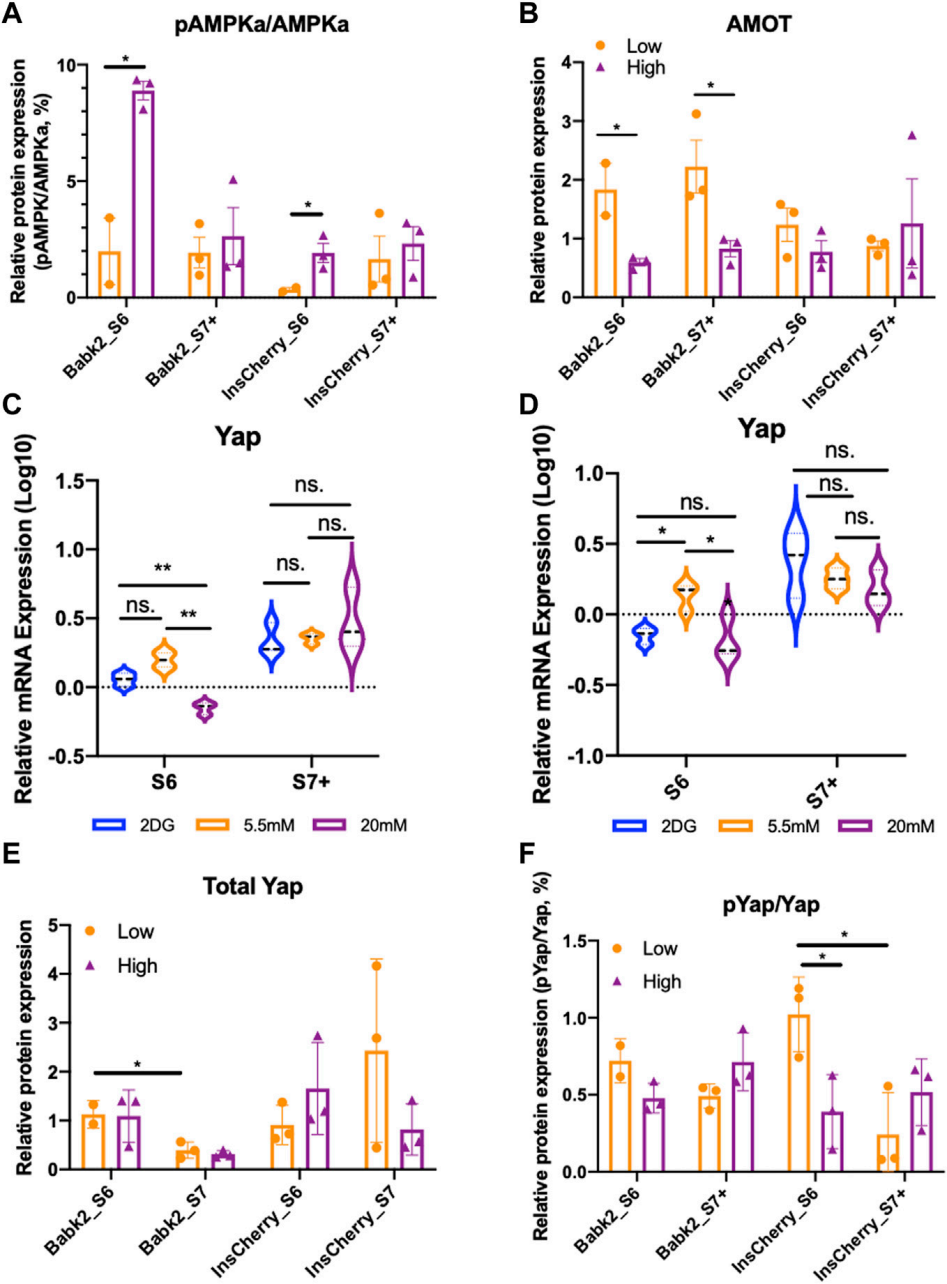
The hippo signaling pathway was transiently regulated by different glucose levels. **(A)** Semi-quantification analysis of pAMPKa to AMPKa protein ratio from Babk2 and InsCherry cell line differentiated under low (5.5 mM) or high (20 mM) glucose conditions (unpaired one-way t-tests). **(B)** Semi-quantification analysis of AMOT protein from Babk2 and InsCherry cell line differentiated in low (5.5 mM) or high (20 mM) glucose conditions (unpaired one-way t-tests). **(C)** *Yap* mRNA expression analysis for Babk2 cell line differentiated cells at different stages (*n* = 3), data were normalized to *TBP* and human islets (*n* = 4), “*Y*-axis = 0” representing the mean value of *Yap* expression in human islets. **(D)** *Yap* mRNA expression analysis for and InsCherry cell line differentiated cells at different stages (*n* = 3). **(E)** Semi-quantification analysis of Yap protein in cells differentiated from Babk2 and InsCherry cell line in low (5.5 mM) or high (20 mM) glucose conditions. **(F)** Semi-quantification analysis of pYap to total Yap protein ratio from Babk2 and InsCherry cell line differentiated in low (5.5 mM) or high (20 mM) glucose conditions. ns, Non-significant, *p < 0.05, **p < 0.01 by unpaired two-way t-tests.

The YAP (Yes-associated protein) activation—Hippo signaling pathway inhibition after the PP stage could facilitate functional beta cell generation [25]. It has been reported that insufficient nutrient inhibits the Hippo signaling via YAP S127 phosphorylation that involves AMPK-mediated regulation of Angiomotin-like 1 (AMOTL1) protein and excludes YAP from the nucleus [26]. The phosphor-AMPKalpha (pAMPKa, phosphor-T172) to AMPKalpha (AMPKa) ratio was significantly higher in cells differentiated under high glucose, whereas total AMPKalpa remains no difference (Figure 4A; Supplementary Figures S7A–C). Due to the basal media without glucose supplementary was not commercially available and prolonged 2-DG supplementary inducing severe cell death during differentiation, we could not further investigate how the pAMPKa/AMPK ratio under a nutrient deprivation condition. However, it has been shown that lactate treatment upregulates the pAMPKa/AMPK ratio [27]. Thus, the upregulation of the pAMPKa/AMPKa might be due to the dramatically higher lactate produced by cells under high glucose conditions rather than the high glucose concentration applied during the differentiation (Supplementary Figure S5C).

AMOT was significantly higher in Babk2-and InsCherry-stage 6 cells differentiated under low glucose (Figure 4B). AMOT family proteins are YAP-binding partners that directly interact with YAP regulation, and AMOTL1 knockdown causes less YAP phosphorylation [28]. Thus, we investigated how glucose variations impact the Hippo by looking at the YAP protein. Babk2-and InsCherry-stage6 cells have a higher YAP expression under low glucose (Figures 4C, D). The total YAP protein did not show a substantial difference regardless of the glucose concentrations applied (Figure 4E; Supplementary Figures S8A, B). However, under low glucose conditions, the phosphorylated-YAP (p-YAP) to total-YAP ratio was higher in InsCherry- and Babk2-stage6 cells (Figure 4F). Thus, we identified a suppressed Hippo signaling pathway activity evidenced by a lower p-YAP/YAP ratio, which involves less stabilized AMOT protein (Figure 4B) under a high glucose condition at stage 6. Meanwhile, the total YAP protein stayed unchanged, suggesting that high glucose differentiation inhibited the Hippo signaling pathway, thus decreasing the IPCs differentiation efficiency in the Babk2 cell line. Of note, in the InsCherry cell line, even though a significant Hippo signaling pathway inhibition in a high glucose condition was found, the IPCs differentiation efficiency was not impacted.

### High Glucose Weakened Mitochondrial Respiration Capacity

**FIGURE 5:**
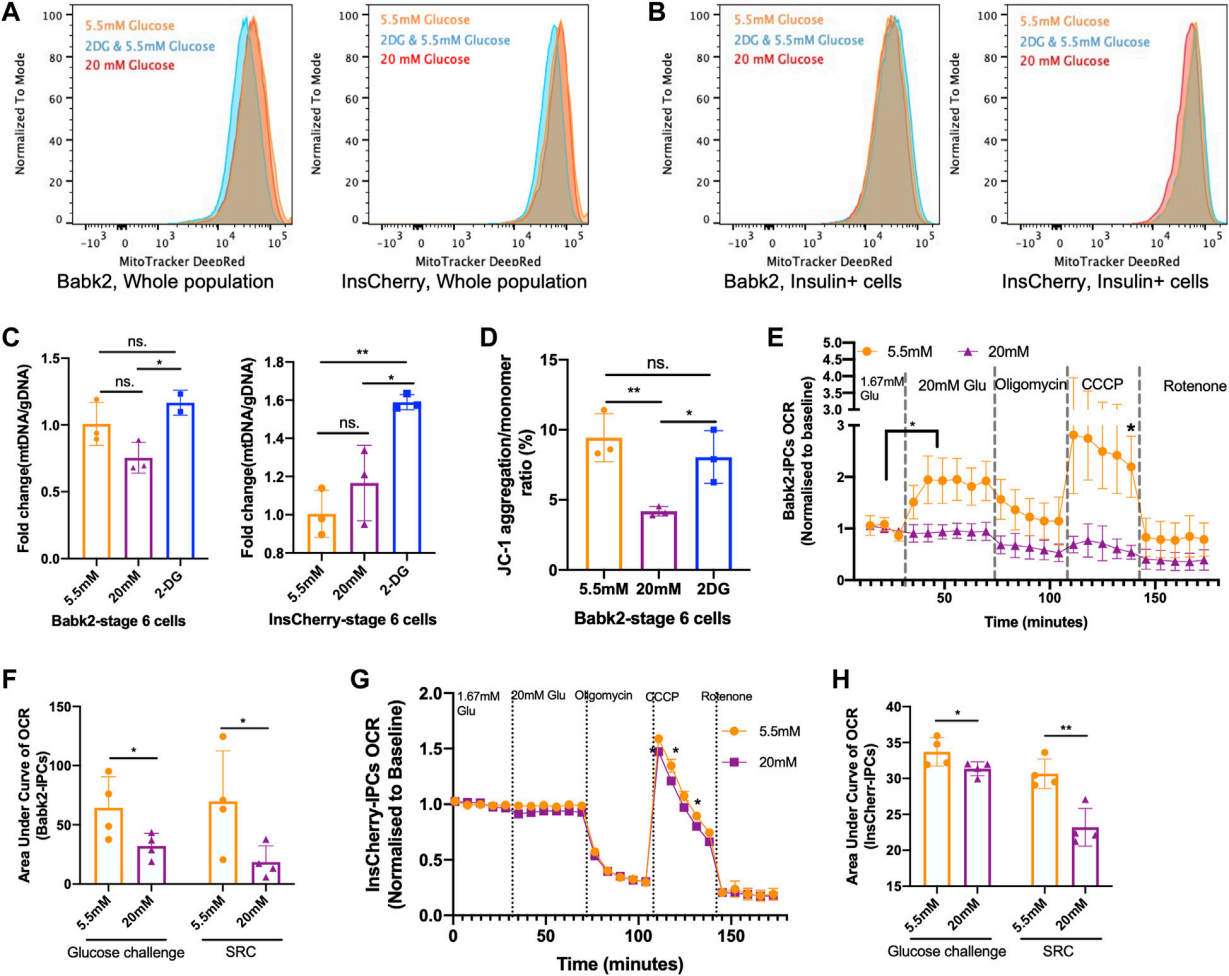
High glucose weakened mitochondrial respiration capacity. **(A)** Flow cytometry assessment of the total mitochondrial contents in Babk2-stage 6 cells (left) and InsCherry-stage 6 cells differentiated at different glucose conditions. **(B)** The mitochondrial contents analysis among insulin + cells in Babk2-stage 6 cells (left) and InsCherry-stage 6 cells (right). **(C)** Mitochondrial DNA (mtDNA) to genomic DNA (gDNA) ratio analysis for Babk2-stage 6 cells (left) and InsCherry-stage 6 cells (right) differentiated at different glucose conditions (*n* = 2–4). **(D)** Flow cytometry quantification of JC-1 aggregation/monomer ratio in Babk2-stage 6 cells differentiated at different glucose conditions (*n* = 3). **(E)** Oxygen consumption rate (OCR) measurements of Babk2-stage 7+ cells under basal conditions and after sequential injections of glucose until final concentration reached 20 mM, Oligomycin 5 μM, CCCP 5 μM, and Rotenone 5 μM, values were normalized to average basal oxygen consumptions (*n* = 4). Data plotted as means ± SEM. **(F)** Area under the curve (AUC) analysis of OCR for Babk2-stage 7+ cells (*n* = 4, unpaired one-way t-tests). SRC, spare respiratory capacity (unpaired one-way *t*-test). **(G)** OCR measurements of InsCherry-stage 7+ cells. Values were normalized to average basal oxygen consumption (*n* = 4). Data plotted as means ± SEM. **(H)** AUC analysis of OCR for InsCherry-stage 7+ cells (*n* = 4, unpaired one-way t-tests). ns, Non-significant, *p < 0.05, **p < 0.01, ***p < 0.001 by unpaired two-way t-tests.

To investigate the effect of glucose levels on the mitochondrial contents during stepwise IPC differentiation, flow cytometry analysis with MitoTacker staining was used. There was no difference in the mitochondrial contents in stage 6 cells under different glucose concentrations (Figure 5A). Mitotracker DeepRed was stained together with an anti-insulin antibody, but there was no significant difference in mitochondrial content among INSULIN + cells in Bbak2-stage 6 cells (Figure 5B, left). However, InsCherry-stage 6 cells under low glucose have more mitochondrial contents, as indicated by the peaks of Mitotracker DeepRed signal slightly right shifted among insulin + cells (Figure 5B, right; Supplementary Figure S9A), suggesting that the INSULIN + cells may have more mitochondrial in number under low glucose differentiation. A higher mtDNA/gDNA ratio was found in Babk2-stage 6 cells differentiated under low glucose but did not show statistical significance (Figure 5C). Furthermore, staining with JC-1, a mitochondrial membrane potential probe, revealed that Babk2-stage 6 cells had significantly higher JC-1 aggregation upon active mitochondria when differentiated under low glucose, which suggests that the mitochondria bioactivity in cells differentiated at high glucose might have been inhibited (Figure 5D; Supplementary Figure S9B).

The oxygen consumption rate (OCR) can better predict islets’ clinical transplantation outcomes in a dose-dependent manner than GSIS [29]. In Babk2-and InsCherry-stage 7+ cells, we found no significant increase in oxygen consumption upon high glucose stimulation (Figures 5E, G). Similar OCR patterns have been reported by others [7, 30]. Opposite, the low glucose-induced Babk2-stage 7+ cells showed a robust oxygen consumption (Figure 5E). InsCherry-stage 7+ cells showed no significant difference in OCR regardless of differentiation under low or high glucose (Figure 5G). The area under the curve (AUC) of the OCR analysis showed that the cells differentiated under low glucose had a significantly higher total oxygen uptake upon glucose challenge, and the spare respiratory capacity was significantly higher in both cells differentiated at a low glucose condition (Figure 5F, H). Our data suggested that the high glucose differentiation weakened the mitochondrial metabolic function.

## Discussion

Hyperglycemia, or high glucose exposure, can adversely affect beta cells [11, 31]. However, the effects of high glucose concentration in *in vitro* beta cell regeneration from hiPSCs have not been studied. By studying the stepwise IPCs differentiation model with three hiPSC lines from different sources, we demonstrated that the effect of glucose concentrations on the IPCs differentiation was cell line dependent, and we unveiled the unintended consequences of high glucose on IPCs differentiation. We showed that the iPS cell line (InsCherry cell lines) benefited from high glucose for IPC differentiation, but the non-pancreatic preferable cell lines (Babk2 and WTC11 cell lines) benefited from low glucose.

In healthy human pregnancies, the fetus’s glucose supply depends on maternal circulation [32]. It is well known that fetal blood glucose levels are usually lower and fluctuate with maternal levels; lower glucose levels correlate with growth-retarded fetal, whereas high blood glucose may cause fetal over-growth [33–36]. In addition, a recent study showed that fetal insulin secretion depends on amino acids rather than glucose [37]. Thus, no evidence has been found *in vivo* that an unphysiologically high glucose level is required for beta cell development. Interestingly, the beta cell maturation process was accelerated when human embryonic stem cell (hESC) derived pancreatic progenitors were exposed to chronic hyperglycemia in mice models [38, 39]. However, in a recently published clinical trial with PP cell transplantation (clinicaltrials.gov: NCT02239354), the enrolled patients were directed to continue comparable insulin therapy to maintain blood glucose well-controlled peri- and post-transplant [40]. In human primary islet transplantation, keeping blood glucose concentrations between 4 and 7 mM peri- and post-transplant is recommended to minimize the loss of islets graft [41]. Thus, relying on high glucose levels to improve beta cell differentiation and maturation may need more concrete evidence, and it seems less practical *in vivo*. Nonetheless, our study revealed that high glucose is required to generate functional beta cells *in vitro* for the IPC differentiation amenable cell line—even though this may represent an artificial condition of the applied protocol and may not replicate the beta cell developmental biology.

Multiple studies have reported that the IPC differentiation efficiency varies from different cell lines [8, 42], thus reducing the flexibility of developing a universal protocol for customized autologous cell transplantation. InsCheery cell line and its parental cell lines have been well studied and characterized previously for IPC differentiation [42, 43]. To the best of our knowledge, WTC11 and Babk2 cell lines have not been used in IPC differentiation before. We recognized that the iPSC shows heterogeneity among cell lines, cells within a line, and temporal states of individual cells [44]. The above variations and complexity of IPC differentiation can influence the findings’ reproducibility and scope of applicability to clinical settings. A dedicated project to investigate the heterogeneity of different clones from single-donor materials generated hiPSC, different donor derived hiPSC, and the comparison with human embryonic stem cells is ongoing and, thus, unable to be covered. Another limitation is the insufficient differentiation efficiency observed at the early stages for Babk2 and WTC11 cell lines, and the significant contributions from the off-target differentiation might dope to our observation even if the same differentiation matrix is applied. Thirdly, we found that an unphysiological high level of glucose (20 mM) was needed for the IPC differentiation amenable cell line differentiation, yet the most suitable glucose concentration remained to be defined. Finally, this study provides essential insight and may raise the attention to glucose concentration during IPC differentiation for future clinical applications, and more investigation is ongoing.

## Data Availability

The original contributions presented in the study are included in the article/Supplementary Material, further inquiries can be directed to the corresponding authors.
