# Supplementary material for: Glucose Concentration in Regulating Induced Pluripotent Stem Cells Differentiation Toward Insulin-Producing Cells
Source: Transpl Int. 2024 Jan 18;37:11900. doi: 10.3389/ti.2024.11900 (PMC10830798; doi:10.3389/ti.2024.11900)
Supplement: Supplementary file 1 [file DataSheet2.PDF]

Supplementary table 1: cell lines information

Name	UCSFi001-A	WTSIi028-A	HMGUi001-A hINS-T2A-H2B-Cherry (+/-)
**Aberration in this study**	WTC11	Babk2	InsCherry*
**Vender**	hPSCreg	hPSCreg	Helmholtz Zentrum München
**Cell line type**	Human induced pluripotent stem cell (hiPSC)		
**Sex**	Male	Female	Female
**Age of donor (at collection)**	30-34	50-54	NA
**Ethnicity**	Asian	White - White British	Caucasian
**Source cell tyoe, origin**	Blood, Derived Peripheral Blood Mononuclear Cell	Zone Of Skin, Fibroblast	Upper Arm, Fibroblast
**Reprogramming Vector type**	Episomal, Non-integrating	Sendai virus, Non-integrating	Episomal, Non-integrating
**Reprogramming Genes**	POU5F1, SOX2, KLF4, MYC, LIN28	POU5F1, SOX2, KLF4, MYC	POU5F1, SOX2, KLF4, MYC, LIN28, NANOG

*Using CRISPR/Cas9, a T2A-H2B-Cherry construct was inserted in one allele of INS exon 3. Information was shown from its parent cell line: HMGUi001-A (hPSCreg Name).

Supplementary table 2: Islets donor characteristics

Islet preparation	**1**	**2**	**3**	**4**	**5**	**6**
Unique identifier	O79	O80	O88	O99	O100	O104
Donor age (years)	49	35	57	57	36	43
Donor sex (M/F)[a]	F	M	M	F	F	M
Donor BMI (kg/m2)	37.6	25.2	24.5	24.2	20.8	32.7
Donor HbA1c (mmol/mol)	34	NA	NA	NA	NA	NA
Origin/source of isletsb	ECIT[b]	ECIT	ECIT	ECIT	ECIT	ECIT
Islet isolation centre	Oslo	Oslo	Oslo	Oslo	Oslo	Oslo
Donor history of diabetes?	No	No	No	No	No	No
For RNA Isolation and analysis	√	√	NA	√	√	NA
For DNA Isolation and analysis	NA	NA	√	√	√	NA
Dynamic GSIS	NA	NA	NA	√	√	√

a. M, Male; F, Female.

b. European Consortium for Islet Transplantation

Supplementary Table 3: Formulation of the differentiation medium

Stage 1 medium: 3days		
Basal medium	Pen/Strep	1%
	$NaHCO_3$	1.174g/L
	Glutamax (100x)	1x
	Glucose	+4.5mM to10mM
	BSA	0.50%
Suplementary	Activin A	100ng/ml
	Chir99021	3μM*
* only added in the first 24 hours		
**Stage 2 medium: 2 days**		
Basal medium	Pen/Strep	1.00%
	$NaHCO_3$	1.174g/L
	Glutamax (100x)	1x
	Glucose	+4.5mM to10mM
	BSA	0.50%
	vitamin C	0.25mM
Suplementary	FGF7	50ng/ml
**Stage 3 medium: 2 days**		
Basal medium	Pen/Strep	1%
	$NaHCO_3$	1.754g/L
	Glutamax (100x)	1x
	Glucose	+4.5mM to10mM
	BSA	2%
	ITS-X	1:200
	vitamin C	0.25mM
Suplementary	Retinoid Acid	1μM
	SANT1	0.25μM
	FGF7	50ng/ml
	LDN193189	0.1μM
	TPB	0.2μM
**Stage 4 medium: 3 days**		
Basal medium	Pen/Strep	1%
	$NaHCO_3$	1.754g/L
	Glutamax (100x)	1x
	Glucose	+4.5mM to10mM
	BSA	2%
	ITS-X	1:200
	vitamin C	0.25mM
Suplementary	Retinoid Acid	0.1μM
	SANT1	0.25μM
	FGF7	2ng/ml
	LDN 193189	0.2μM
	TPB	0.1nM

Stage 5 medium: 3 days		
Basal medium	Pen/Strep	1%
	$NaHCO_3$	1.754g/L
	Glutamax (100x)	1x
	Glucose	Final conc. at 5.5mM or 20mM
	BSA	2%
	ITS-X	1:200
	Heparin	10ug/ml
	vitamin C	0.25mM
	$ZnSO_4$	10μM
Suplementary	Retinoid Acid	0.05μM
	SANT1	0.25μM
	ALK5 inh II	10μM
	T3	1μM
	LDN 193189	0.1μM
**Stage 6 medium: 7 days**		
Basal medium	Pen/Strep	1%
	$NaHCO_3$	1.754g/L
	Glutamax (100x)	1x
	Glucose	Final conc. at 5.5mM or 20mM
	BSA	2%
	ITS-X	1:200
	Heparin	10ug/ml
	vitamin C	0.25mM
	$ZnSO_4$	10μM
Suplementary	ALK5 inh II	10μM
	T3	1 μM
	LDN	100nM
	Xxi	0.1μM
**Stage 7 medium: 7days**		
Basal medium	Pen/Strep	1%
	$NaHCO_3$	1.754g/L
	Glutamax (100x)	1x
	Glucose	Final conc. at 5.5mM or 20mM
	BSA	2%
	ITS-X	1:200
	Heparin	10ug/ml
	vitamin C	0.25mM
	$ZnSO_4$	10μM
Suplementary	ALK5 inh II	10μM
	T3	1 uM
	N-acetyl cysteine	1mM
	Trolox	10μM
	R428	2μM
**Stage 7+: 7-10 days**		
Same medium as in Stage 7, cells were dissociated and resuspened in Ultra Low Attachment plates at ~1*10^6 cells/ml and maintained in orbital shaker (90RPM) in 5% CO2, 37℃ incubator.		

Supplementary Table 4: List of antibodies

Antibody	Catalog No.	Vendor	Dilution factor	Application[1]
Guinea Pig anti-insulin	A0564	Dako	1:500	IF,FCS
Goat anti Human PDX-1	AF2419	R&D Systems	1:200	IF,FCS
Mouse anti Nkx-6.1	F55A12-S	University of Iowa,DHB	1:100	IF,FCS
Mouse anti YAP1 Antibody (63.7)	SC-101199	Santa Cruz	1:1000	WB
Rabbit anti Phospho-YAP (Ser127)	4911	Celll Signaling Technology	1:1000	WB
Mouse anti Angiomotin (AMOT, B-4)	SC-166924	Santa Cruz	1:500	WB
Mouse anti AMPKalpha	2793S	Celll Signaling Technology	1:1000	WB
Rabbit p-AMPKalpha (40H9)(T172)	2535S	Celll Signaling Technology	1:1000	WB
Rabbit anti actin	A2066	Sigma-Aldrich	1:5000	WB

[1]IF: Immunofluorescence; WB: Western blot; FCS: Flow cytometry

Supplementary Table 5: List of oligonucleotides for qRT-PCR

**Gene name**	**Forward primer sequence**	**Reverse primer sequence**
TBP	TGTATCCACAGTGAATCTTGGTTG	GGTTCGTGGCTCTCTTATCC
SOX9	GATTAGCACACTGATCACACGA	TTAACCCTCTTCAGAGCAAGC
NGN3	CTAAGAGCGAGTTGGCACTG	TGCCGAGTTGAGGTTGTGCA
PAX4	GGCAGTATCCTGATTCAGTGGC	CTTCTCTTGCCGACGCCATTT
ARX	CTGCTGAAACGCAAACAGAGG	CTCGGTCAAGTCCAGCCTCAT
NEUROD1	ATCAGCCCACTCTCGCTGTA	GCCCCAGGGTTATGAGACTAT
NKX2-2	GGAGCTTGAGTCCTGAGGG	TCTACGACAGCAGCGACAAC
PDX1	CGTCCGCTTGTTCTCCTC	CCTTTCCCATGGATGAAGTC
NKX6.1	CTATTCGTTGGGGATGACAGAG	TGTCTCCGAGTCCTGCTTCT
INS	ACGAGGCTTCTTCTACACACC	TCCACAATGCCACGCTTCTG
GCG	CGTTCCCTTCAAGACACAGAG	GCCTGGAGTCCAGATACTTG
SST	TGGGTTCAGACAGCAGCTC	CCCAGACTCCGTCAGTTTCT
MAFA	GAGAGCGAGAAGTGCCAACT	TTCTCCTTGTACAGGTCCCG
MAFB	ACGCCTACAAGGTCAAGTGC	CGACTCACAGAAAGAACTCGG
ISL1	TCACGAAGTCGTTCTTGCTG	CATGCTTTGTTAGGGATGGG
UCN3	GGAGGGAAGTCCACTCTCG	TGTAGAACTTGTGGGGGAGG
LDHA	GGCCTGTGCCATCAGTATCT	GGAGATCCATCATCTCTCCC
ABCC8	GAGAAGTCGGCCTCTTTGAA	GGGCCTTTGCCATCTATACC
YAP	TGTCCCAGATGAACGTCACAG	TGGTGGCTGTTTCACTGGAG
VDAC1	GCAAAATCCCGAGTGACCCA	TCCAGGCAAGATTGACAGCG
VDAC2	CTTTGCAGTGGGCTACAGGA	GAGTGCAGTTGGTACCTGATG
GLUT1	GCAGGCTTCTCCAACTGGAC	GAACCAGGAGCACAGTGAAG
Mdh1	GGTGTCCTAATGGAACTGCAAG	TCCAGGTCTTTGAAGGCAACG
PSCK1	CCAGATGTGCAGGAGAAATTGC	CGTCACAATGCCATCCAGCAT
ß2M	TGCTGTCTCCATGTTTGATGTATCT	TCTCTGCTCCCCACCTCTAAGT
mtDNA 16S rRNA	GCCTTCCCCCGTAAATGATA	TTATGCGATTACCGGGCTCT
