# Supplementary material for: Glucose Concentration in Regulating Induced Pluripotent Stem Cells Differentiation Toward Insulin-Producing Cells
Source: Transpl Int. 2024 Jan 18;37:11900. doi: 10.3389/ti.2024.11900 (PMC10830798; doi:10.3389/ti.2024.11900)
Supplement: Supplementary file 2 [file DataSheet1.PDF]

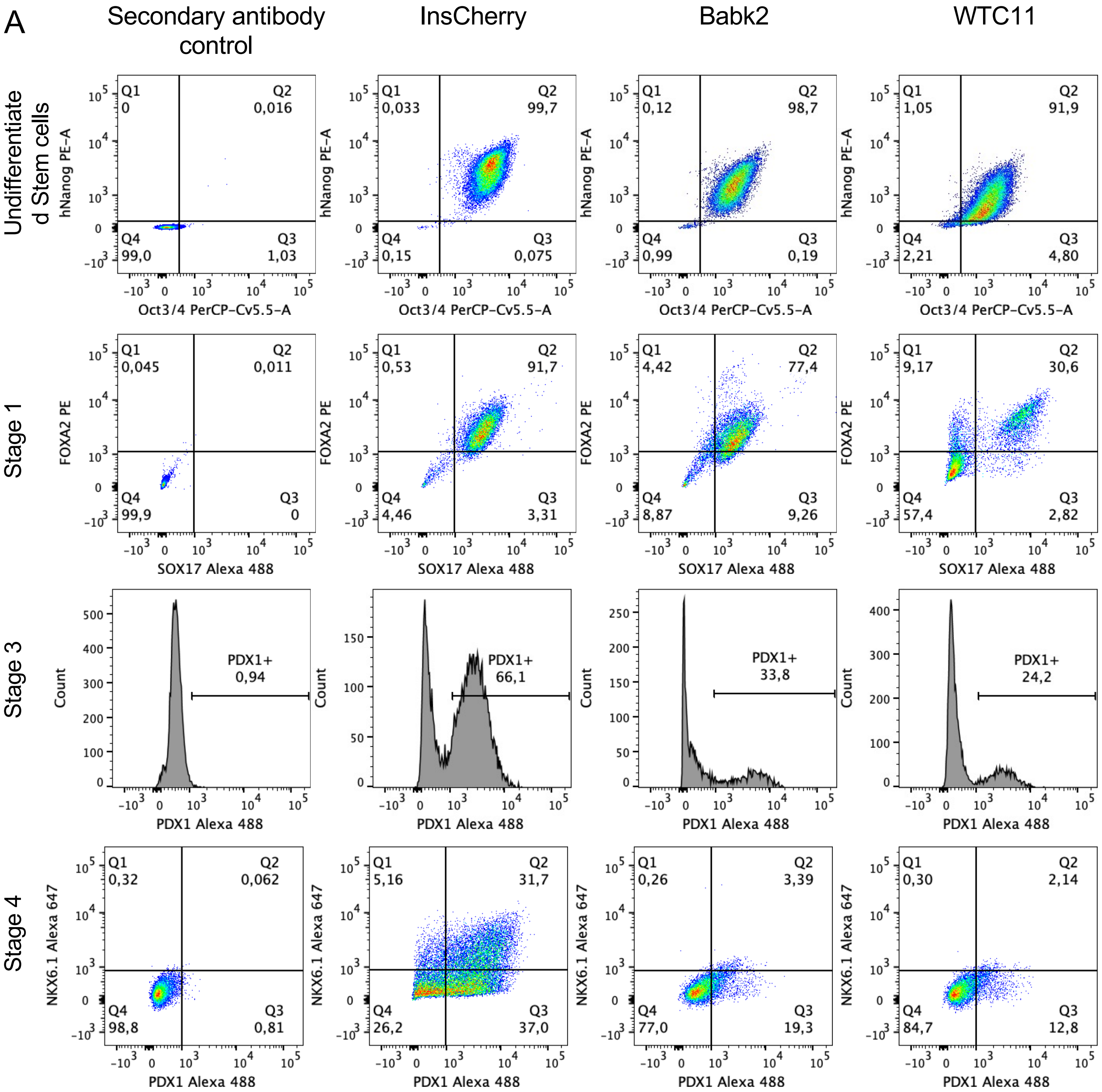

Supplementary Figure S1.
(A) Representative flow cytometry plots for InsCherry, Babk2, and WTC11 cell line differentiation from undifferentiated cells towards stage 4 cells.

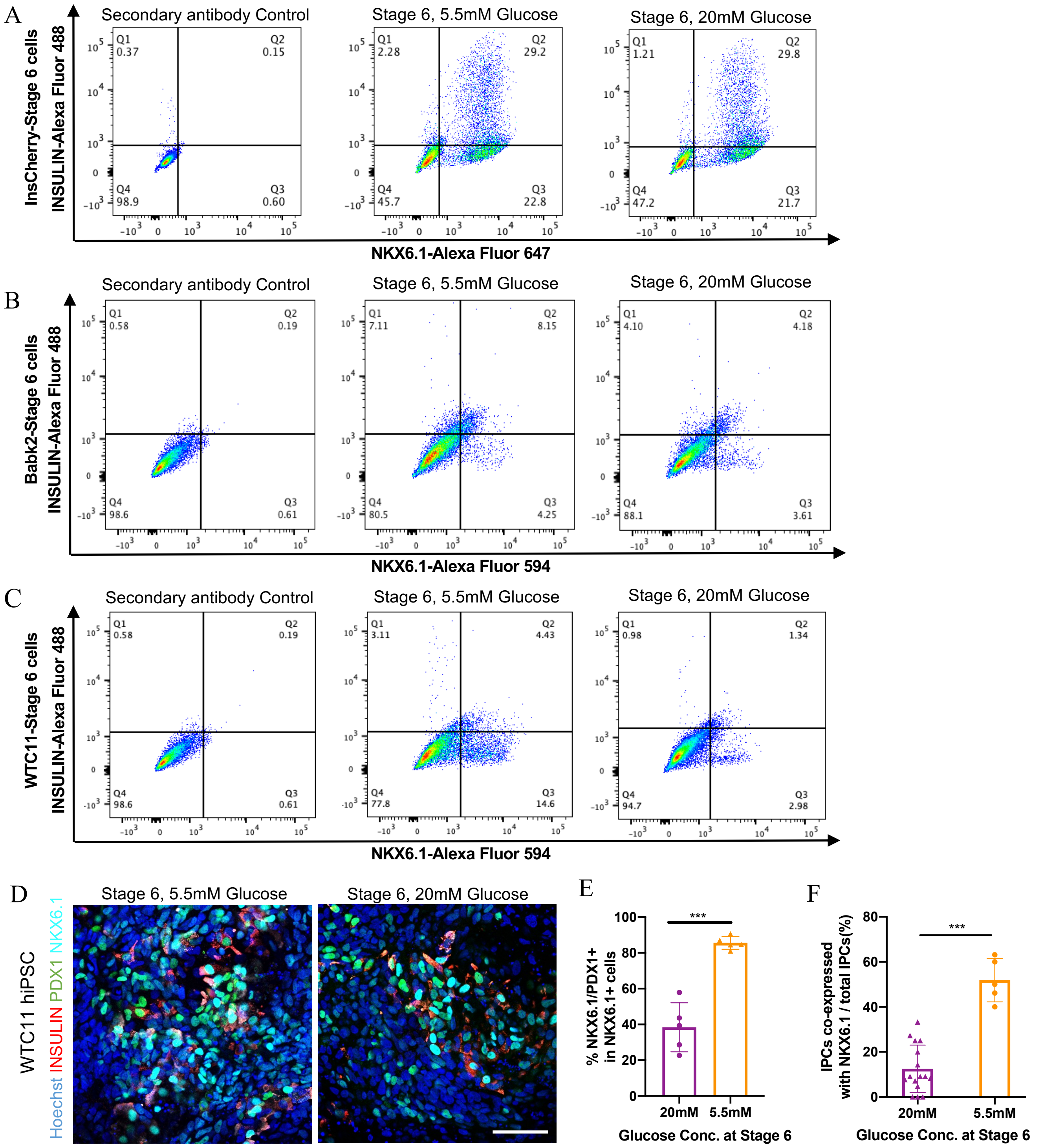

Supplementary Figure S2.

(**A**) Representative flow cytometry plots for InsCherry-Stage 6 cells staining for INSULIN and NKX6.1. (**B**) Representative flow cytometry plots for Babk2-Stage 6 cells staining for INSULIN and NKX6.1. (**C**) Representative flow cytometry plots for WTC11-Stage 6 cells staining for INSULIN and NKX6.1. (**D**) Immunostaining of INSULIN, PDX1 and NKX6.1 for WTC11-Stage 6 cells. (**E**) PDX1 and NKX6.1 co-localization percentage among NKX6.1+ cells in WTC11-Stage 6 cells, n=5. (**F**) Percentage of INSULIN/NKX6.1+ IPCs among INSULIN+ IPCs in Babk2-Stage 6 cells. Scale bars represent 50μm; ***P<0.001 by unpaired two-way t-tests.

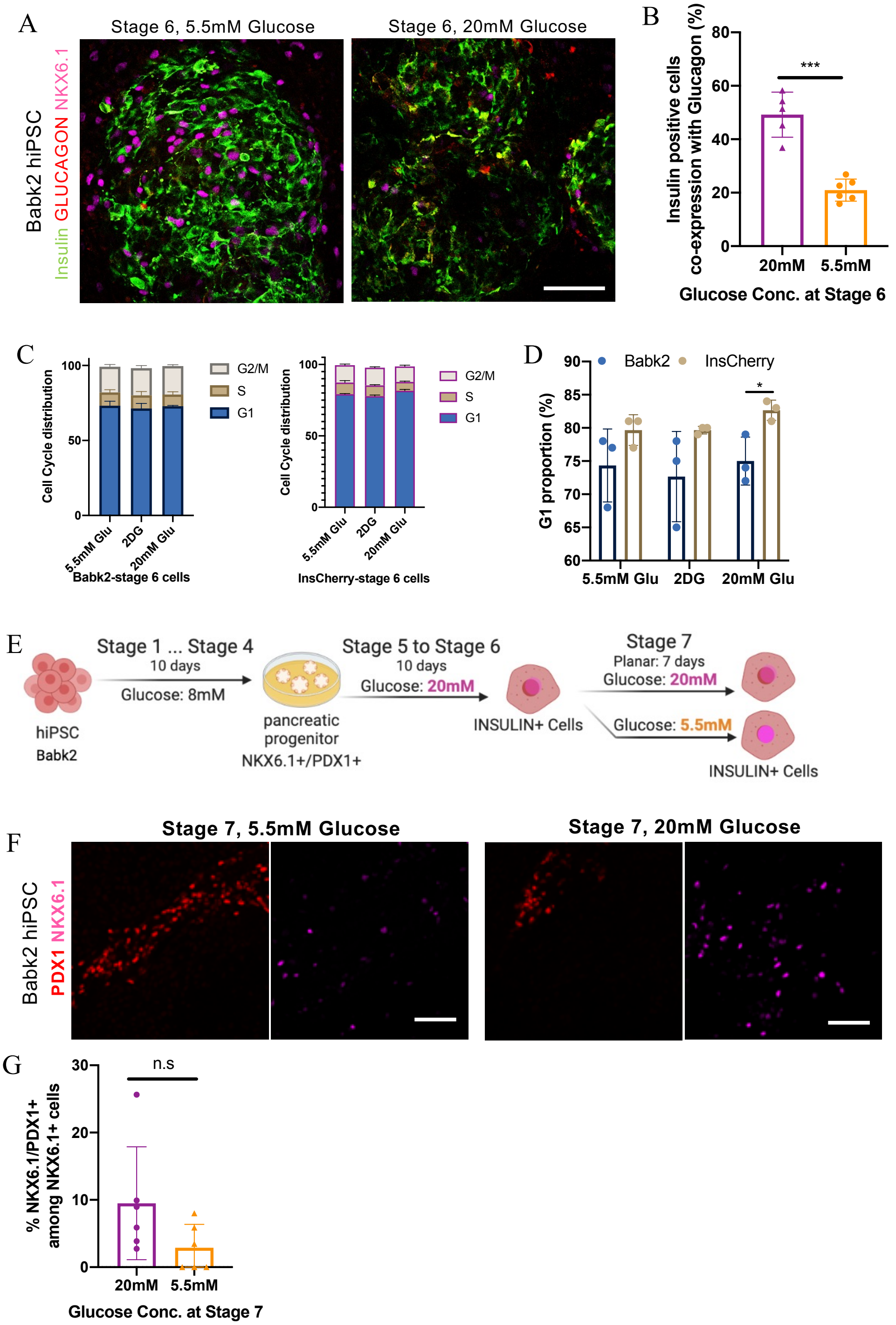

Supplementary Figure S3.
(**A**) Immunostaining of INSULIN, GLUCAGON, and NKX6.1 for Babk2-stage 6 cells. (**B**) Quantitative image analysis for INSULIN/GLUCAGON+ cells among INSULIN+ cells for Babk2-stage 6 cells differentiated under different glucose conditions, n>=5. (**C**) Cell cycle distribution of Babk2-stage 6 cells (left) and InsCherry-stage 6 cells (right), n=3. (**D**) G1 proportion of cell cycle analysis for Babk2-stage 6 and InsCherry-stage 6 cells differentiated under different glucose conditions, n=3. (**E**) Outline of the experiment design: cells after the pancreatic progenitor stage (stage 4) were cultured in stage 5 and 6 medium containing 20 mM of glucose for 10 days, and the cells were then entered to stage 7 medium containing 5.5 mM or 20 mM glucose. (**F**) Immunostaining of PDX1 and NKX6.1 for Babk2-stage 7 cells cultured at 5.5 mM or 20 mM glucose. (**G**) The percentage of NKX6.1/ PDX1+ cells among NKX6.1+ cells in Babk2-stage 7 cells, which have been cultured at 20 mM glucose during stage 5 and 6, and incubated in 5.5mM or 20mM of glucose at stage 7, n=6. Scale bars represent 50 μm; ns. Non-significant, *p<0.05, ***P<0.001 by unpaired two-way t-tests.

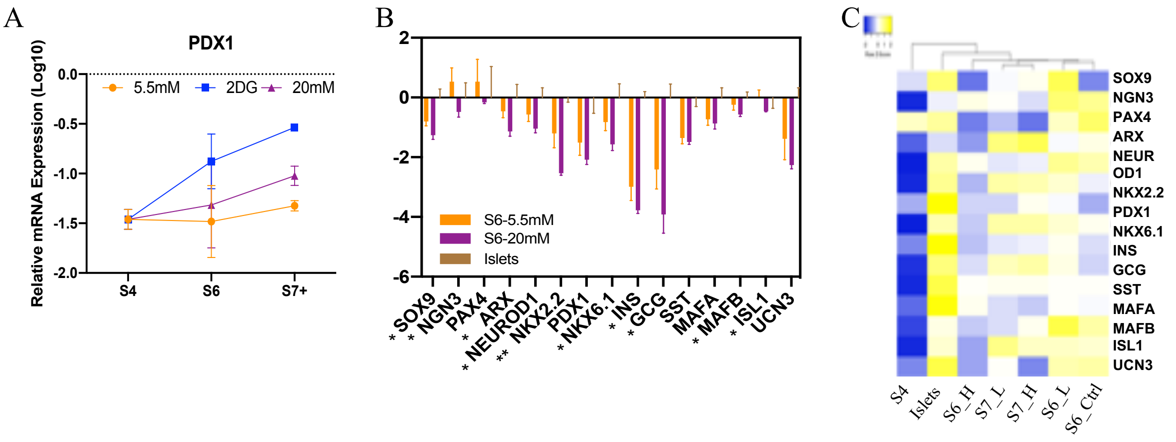

Supplementary Figure S4.
(**A**) *PDX1* gene expression analysis of Babk2 cell line differentiated cells at different stages (n=3). Data were normalized to *TBP* and then human islets (n=4), "Y-axis = 0" representing the mean value of each gene expression in human islets. (**B**) Endocrine-related gene expression profile of WTC11-stage 6 cells differentiated at different glucose conditions. (**C**) Heatmap of 15 endocrine-related gene expression profiles in cells differentiated from Babk2 cell line at different stages. "L," 5.5 mM low glucose differentiation medium; "H," 20 mM high glucose differentiation medium, "Ctrl," osmotic control group by supplementing 14.5 mM mannitol in the 5.5 mM glucose differentiation medium. ns. Non-significant, * P<0.05, **P<0.01 by unpaired two- way t-tests.

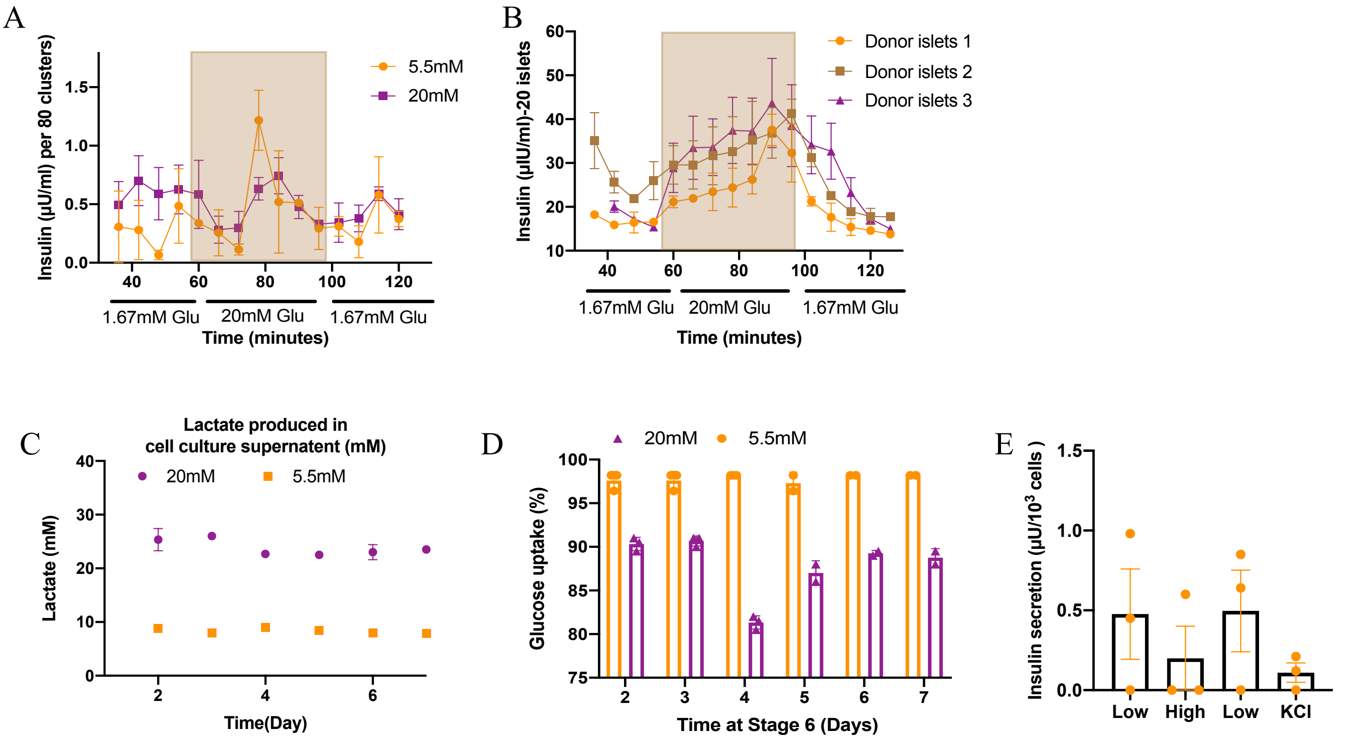

Supplementary Figure S5.
(**A**) Dynamic GSIS of InsCherry-stage 7+ cells from low (5.5 mM) and high (20 mM) glucose conditions (n=3). Data plotted with means ± SEM. (**B**) Dynamic GSIS of human primary islets (n=3, 20 islets each). Data plotted with means ± SEM. (**C**) Lactate productions in daily cell culture supernatant during stage 6 of Babk2 cell line differentiation (n=3). (**D**) The daily glucose uptake percentage during stage 6 of Babk2 cell line differentiation (n=3). (**E**) GSIS of WTC11-stage 7+ cells differentiated under a low (5.5 mM) glucose condition, cells were challenged with low glucose (1.67 mM), high glucose (20 mM), low glucose (1.67 mM) for 60 minutes, and KCl (30 mM) for 30 minutes, n=3. ns. Non-significant, * P<0.05, **P<0.01 by unpaired two-way t-tests.

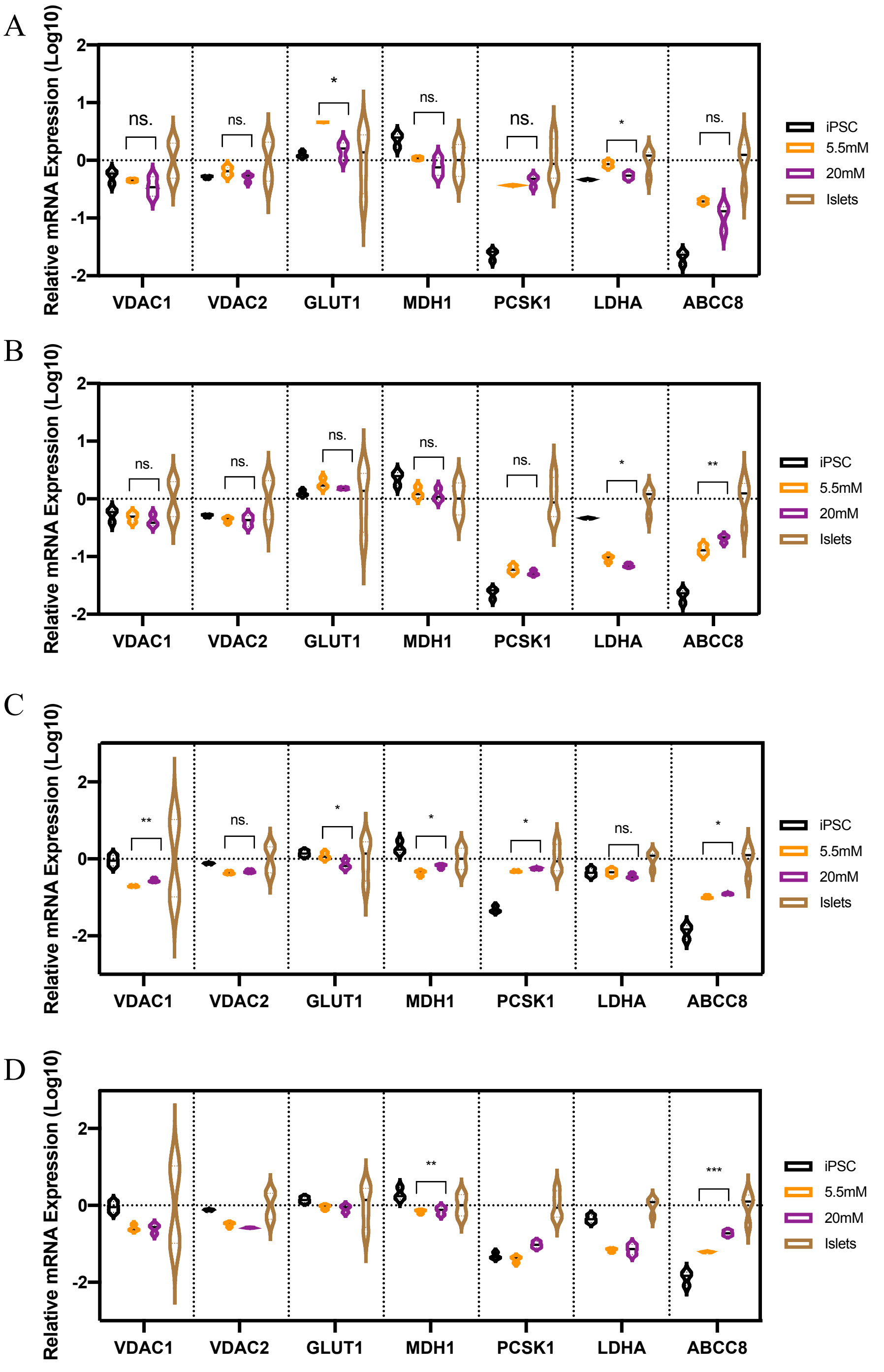

Supplementary Figure S6.
(**A**) and (**B**) Metabolism related genes expression analysis for Babk2-stage 6 cells (A) and Babk2-stage 7+ cells (B), n=3. (**C**) and (**D**) Metabolism related genes expression analysis for InsCherry-stage 6 cells (C) and InsCherry-stage 7+ cell (D), n=3. Data normalized to *TBP* and then normalized to the gene expression value in human islets. ns. Non-significant, * P<0.05, **P<0.01, ***P<0.001 by unpaired two-way t-tests.

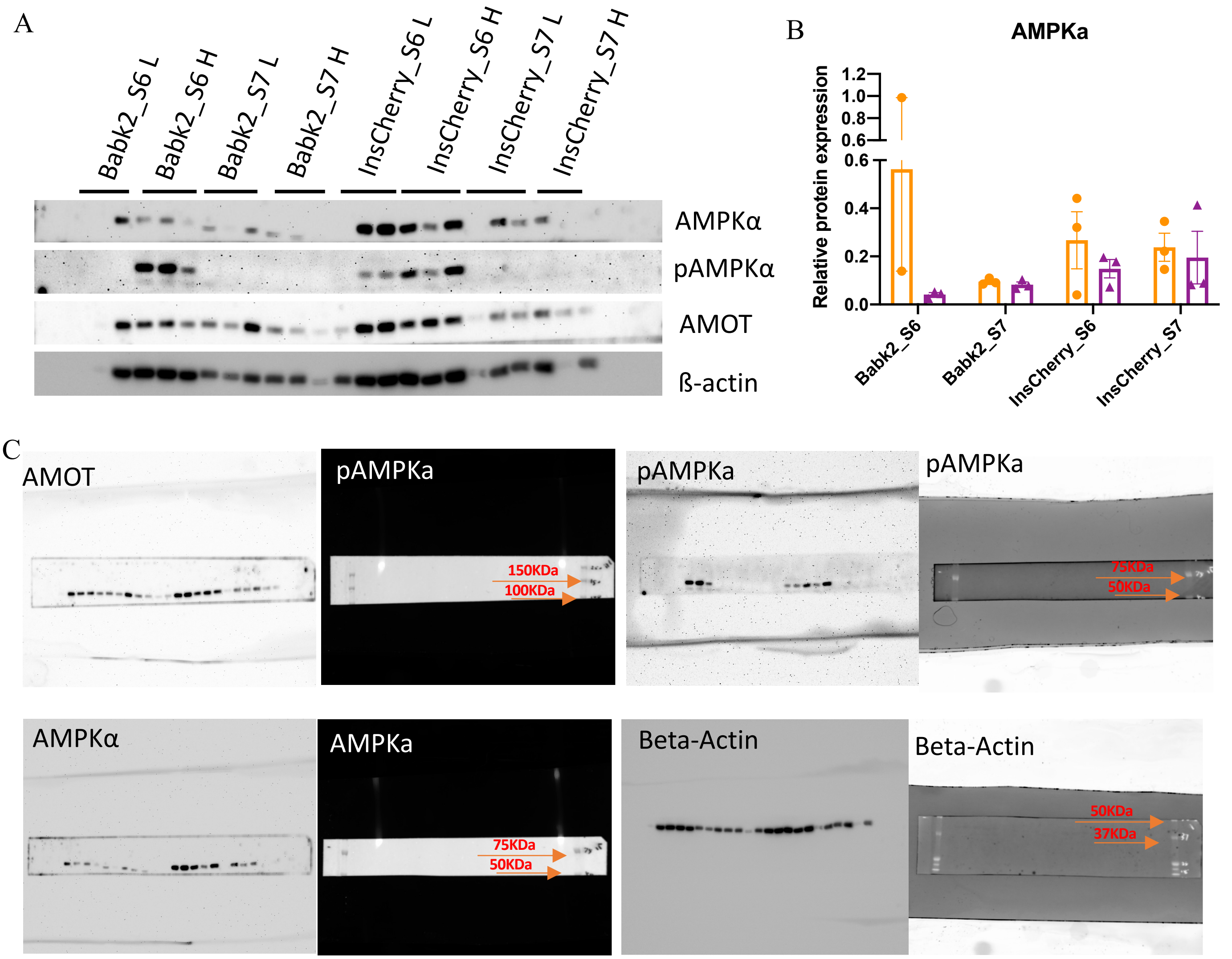

Supplementary Figure S7.
(**A**) Western blots analysis of AMPK signaling pathway at stage 6 and stage 7+ from Babk2 and InsCherry cell line. (**B**) Semi-quantification analysis of total AMPKa protein from Babk2 and InsCherry cell line differentiated under low (5.5 mM) or high (20 mM) glucose conditions (unpaired one-way t-tests) (**C**) Raw images of western blots analysis.

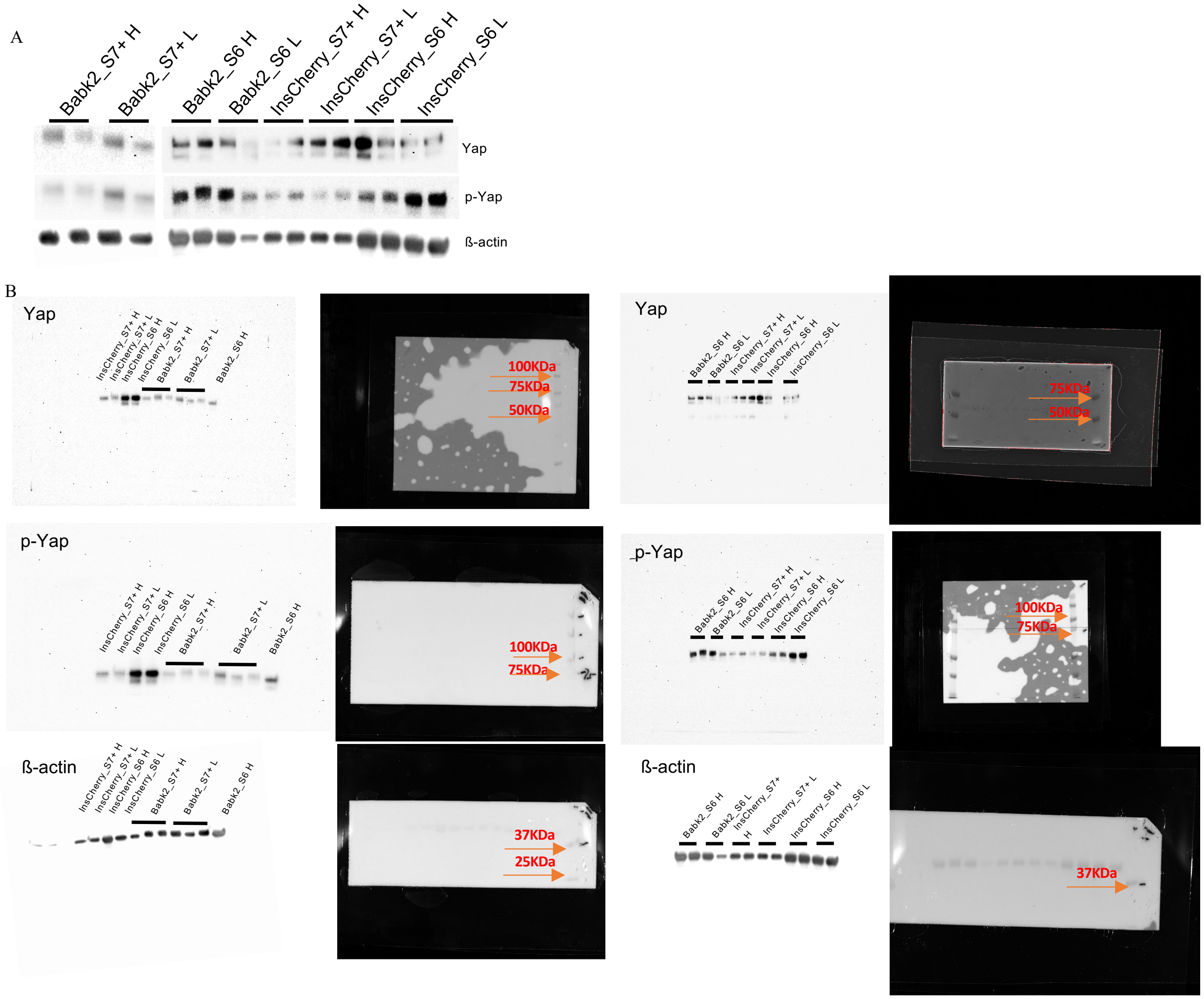

Supplementary Figure S8.
(**A**) Western blots analysis of Yap and p-Yap within differentiated cells at stage 6 and stage 7+ from Babk2 and InsCherry cell line (n=2). (**B**) Raw images of western blots analysis.

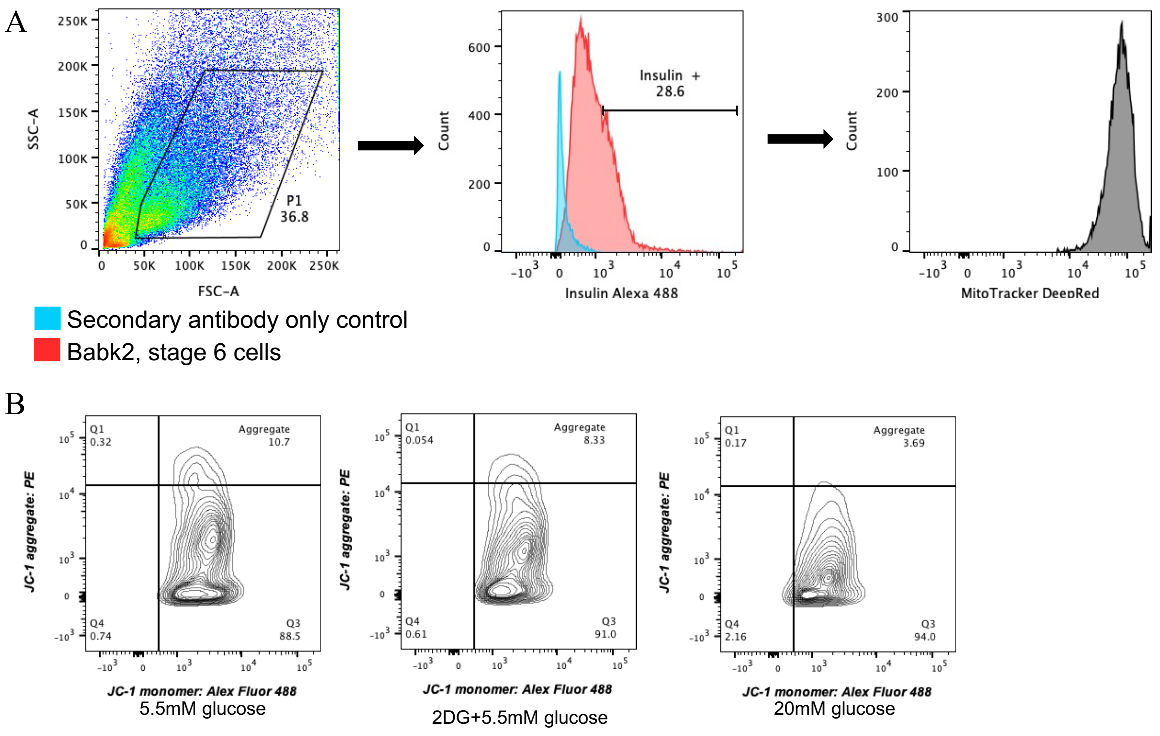

Supplementary Figure S9.
(**A**) Gating strategies for mitochondrial contents analysis in stage 6 cells and among their insulin-positive cells. Cells were double stained with MitoTracker DeepRed and INSULIN. Blue shows secondary antibody only control, and red shows Babk2-stage 6 cells. (**B**) Flow cytometry quantification of JC-1 aggregation upon active mitochondria in Babk2-stage 6 cells differentiated at different glucose conditions.
